# Exercise‐Induced Extracellular Vesicles as Mediators of Mitochondrial Biogenesis and Insulin Sensitivity in Metabolic Adaptation

**DOI:** 10.1002/edm2.70257

**Published:** 2026-06-14

**Authors:** Jian Wang, Lei Wu

**Affiliations:** ^1^ Xinjiang Vocational University Urumqi City Xinjiang China; ^2^ The Affiliated Hospital to Changchun University of Chinese Medicine Changchun Jilin China

**Keywords:** exercise, extracellular vesicles, insulin sensitivity, metabolic homeostasis, mitochondrial biogenesis

## Abstract

**Background:**

Metabolic diseases like type 2 diabetes and obesity share insulin resistance as a common feature, driven partly by mitochondrial dysfunction. Exercise‐induced extracellular vesicles (EVs) have emerged as mediators of inter‐organ communication in metabolic regulation.

**Objective:**

To synthesize evidence on exercise‐induced EVs in mitochondrial adaptation and insulin sensitivity, and propose an integrative framework linking EV‐mediated communication to systemic metabolic benefits:

**Methods:**

Narrative synthesis of mechanistic, animal, EV transfer/inhibition, translational, and human studies.

**Results:**

Exercise alters EV abundance and cargo, including mitochondrial proteins, metabolic enzymes, and microRNAs. These cargoes may activate energy‐sensing, NAD^+^‐dependent, transcriptional, and post‐transcriptional pathways to enhance mitochondrial biogenesis and oxidative metabolism. By improving substrate utilization and reducing lipotoxicity, mitochondrial ROS, ER stress, and inflammation, EV‐mediated mitochondrial adaptation may boost insulin sensitivity and insulin signaling. EV transfer/inhibition studies support a contributory role for exercise‐induced EVs in glucose homeostasis, though evidence remains context‐dependent.

**Conclusions:**

Exercise‐induced EVs may link exercise stimuli to mitochondrial adaptation and systemic insulin sensitivity. The proposed “exercise–EV–mitochondrial adaptation–insulin sensitivity” framework offers a conceptual basis for understanding systemic metabolic adaptation and highlights translational potential for metabolic diseases. Future work should clarify causality, tissue specificity, pharmacokinetics, and standardization.

## Graphical abstract

Exercise stimulates the release of extracellular vesicles from metabolically active tissues. These vesicles transport regulatory cargos, including microRNAs, proteins, lipids, and metabolites, to target organs such as skeletal muscle, adipose tissue, liver, and vascular tissues. Through intercellular communication, exercise‐induced EVs regulate mitochondrial biogenesis, oxidative metabolism, glucose uptake, and insulin signaling, thereby contributing to systemic metabolic adaptation and improved insulin sensitivity.

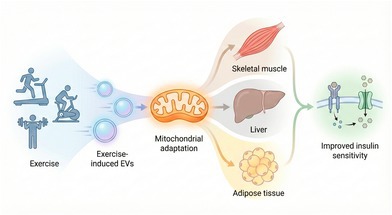

## 1 Introduction

Metabolic diseases, including type 2 diabetes, obesity, and non‐alcoholic fatty liver disease, remain highly prevalent worldwide, with insulin resistance recognized as their common pathological hallmark [1]. Insulin resistance is typically accompanied by impaired insulin signalling, dysregulated lipid metabolism, and chronic low‐grade inflammation [2, 3]. It is also associated with a markedly increased risk of cardiovascular complications. Accumulating evidence suggests that mitochondrial dysfunction, particularly impaired mitochondrial biogenesis, is closely associated with reduced insulin sensitivity and may contribute to its development and maintenance in a context‐dependent manner [4]. In obesity and type 2 diabetes, mitochondrial oxidative phosphorylation efficiency is decreased, fatty acid β‐oxidation is impaired, ATP production is reduced, and reactive oxygen species accumulate [4, 5]. These alterations may aggravate energy metabolic imbalance and defective insulin signal transduction, although they may also reflect compensatory adaptations to nutrient excess or other metabolic stresses under certain conditions [3]. Accordingly, targeting mitochondrial quality, metabolic flexibility, and mitochondrial biogenesis has been considered a potential strategy for improving insulin sensitivity.

Regular exercise is widely acknowledged as an effective non‐pharmacological intervention for improving insulin sensitivity and metabolic health [6]. Its classical mechanisms have been largely attributed to the activation of energy‐sensing pathways and enhanced mitochondrial biogenesis within skeletal muscle [7]. However, exercise‐induced metabolic adaptation likely reflects the combined effects of tissue‐autonomous responses and systemic inter‐organ communication. Although local energy‐sensing pathways remain central, they cannot fully explain the coordinated mitochondrial remodelling across skeletal muscle, liver, and adipose tissue or the systemic improvement in whole‐body metabolic homeostasis. These observations suggest that exercise‐induced metabolic benefits involve inter‐organ communication mechanisms in addition to signalling events within individual tissues [8]. In this context, extracellular vesicles (EVs) have emerged as important mediators of intercellular and inter‐organ communication [9]. Exercise stimulates the release of circulating EVs and reshapes their cargo profiles. These EVs can transmit integrated metabolic signals to multiple target tissues. Exercise‐induced EVs have been implicated in tissue repair, inflammatory regulation, and the maintenance of metabolic homeostasis [10]. They may also enhance insulin sensitivity in skeletal muscle, adipose tissue, and liver by modulating glucose transport, lipid metabolism, and mitochondrial function. Nevertheless, most existing studies have focused on individual tissues or single experimental models [11]. A coherent inter‐organ framework explaining how exercise‐induced EVs coordinate mitochondrial biogenesis and systemic insulin sensitivity is still lacking [12].

In this review, we synthesize recent advances in the role of exercise‐induced EVs as inter‐organ signalling mediators in metabolic regulation, with a focus on their coordinated control of mitochondrial biogenesis and insulin sensitivity. By integrating molecular mechanisms, in vivo functional evidence, and emerging translational studies, we propose an integrative “exercise–EV–mitochondrial adaptation–insulin sensitivity” framework to conceptualize how EV‐mediated inter‐organ communication amplifies the systemic metabolic benefits of exercise. The seminal work by Whitham et al. established exercise‐induced EVs as important mediators of tissue crosstalk during exercise and provided a foundational framework for understanding EV‐based systemic communication [13]. Building on this work, the present review focuses specifically on how exercise‐induced EVs regulate mitochondrial adaptation and insulin sensitivity in metabolic tissues. Unlike previous reviews that mainly emphasized EV release, cargo profiling, and general inter‐organ communication during exercise, we integrate evidence linking EV cargoes with mitochondrial biogenesis, oxidative metabolism, and insulin signalling. In particular, we highlight mitochondrial adaptation as a mechanistic bridge through which EV‐mediated inter‐organ communication may translate exercise stimuli into improved insulin sensitivity. By doing so, we propose an integrative “exercise–EV–mitochondrial adaptation–insulin sensitivity” framework for understanding metabolic disease.

## 2 Mitochondrial Dysfunction in Insulin Resistance

### 2.1 Mitochondrial Biogenesis and Insulin Sensitivity

Mitochondrial biogenesis refers to the process by which cells generate new mitochondria through the coordinated expression of nuclear and mitochondrial genomes. This process maintains mitochondrial content and functional homeostasis. Together with mitochondrial dynamics and selective mitophagy, mitochondrial biogenesis forms the core of the mitochondrial quality control network and plays an important role in cellular energy homeostasis [14]. Under conditions of increased metabolic demand or limited energy supply, activation of mitochondrial biogenesis enhances oxidative phosphorylation, promotes fatty acid oxidation, and alleviates metabolic stress [15]. These adaptive responses support efficient energy production and metabolic flexibility.

Peroxisome proliferator‐activated receptor‐γ coactivator 1α (PGC‐1α) is widely recognized as a central regulator of mitochondrial biogenesis [16]. PGC‐1α cooperates with nuclear respiratory factors 1 and 2 (NRF1 and NRF2) and mitochondrial transcription factor A (TFAM) [17, 18]. Together, these factors promote the transcription of nuclear‐encoded mitochondrial proteins and the replication and transcription of mitochondrial DNA. This coordinated program increases mitochondrial number and improves mitochondrial function. Extensive evidence suggests that the PGC‐1α signalling axis regulates mitochondrial biogenesis and substrate oxidation in metabolic organs such as skeletal muscle, liver, and adipose tissue [19]. Alterations in PGC‐1α activity influence whole‐body energy metabolism and insulin sensitivity [20].

In insulin resistance–related states, mitochondrial biogenesis is often suppressed. This suppression is reflected by reduced PGC‐1α expression or activity, decreased mitochondrial DNA copy number, and impaired oxidative capacity. These changes may contribute to lipid accumulation, energy metabolic imbalance, and defective insulin signalling. However, the relationship between mitochondrial adaptation and insulin sensitivity is complex and context‐dependent [18, 20]. For example, high‐fat diet–induced insulin resistance can be accompanied by mitochondrial adaptations that partially resemble exercise‐induced remodelling, suggesting that increased mitochondrial capacity does not necessarily translate into improved insulin action. In this setting, mitochondrial changes may represent compensatory responses to nutrient excess rather than direct drivers of insulin resistance. Therefore, mitochondrial dysfunction should be viewed as one component of a broader multifactorial network involving lipid intermediates, inflammatory signalling, oxidative stress, nutrient overload, and tissue‐specific defects [21]. Accordingly, restoring mitochondrial quality and metabolic flexibility, rather than simply increasing mitochondrial biogenesis, may represent a more balanced strategy for improving insulin sensitivity.

### 2.2 Systemic Patterns of Mitochondrial Dysfunction

Metabolic disturbances associated with insulin resistance typically involve multiple organs. Although mitochondrial dysfunction shows tissue‐specific features, it displays similar patterns of metabolic imbalance at the systemic level [22]. In skeletal muscle, insulin resistance is more accurately characterized by impaired metabolic flexibility than by a uniform decrease in fatty acid oxidation across all conditions [23]. Under fasting or resting conditions, reduced mitochondrial oxidative capacity and incomplete fatty acid oxidation may promote the accumulation of lipid intermediates. However, during exercise or insulin‐stimulated states, insulin‐resistant individuals may continue to rely disproportionately on lipid oxidation and show reduced switching toward carbohydrate utilization [24]. For example, in adults with impaired glucose tolerance, impaired fasting glucose has been shown to shift fuel selection toward increased fat oxidation and decreased muscle glycogen utilization during exercise. Thus, mitochondrial dysfunction in insulin resistance should be understood as a state‐dependent defect in substrate switching, rather than simply as reduced fat oxidation. These abnormalities may interfere with insulin signaling and glucose utilization, particularly when accompanied by nutrient overload, lipid intermediates, and inflammatory stress.

In the liver, limited mitochondrial fatty acid oxidation and insufficient ATP production may promote lipid deposition and enhance inflammatory responses [25]. In adipose tissue, decreased mitochondrial density and oxidative capacity, together with impaired thermogenic function, may reduce whole‐body energy expenditure [26]. Despite occurring in different tissues, these alterations converge on a common phenotype characterized by impaired mitochondrial quality control, reduced metabolic flexibility, and disrupted substrate handling. Importantly, cross‐tissue mitochondrial dysfunction does not occur in isolation [27]. It is amplified by metabolic stress, inflammatory signalling, and lipid mediators. These processes sustain insulin resistance at the systemic level. Thus, insulin resistance should not be viewed as a disorder of a single organ. Instead, it represents a systemic metabolic disease involving maladaptive mitochondrial remodelling, lipid overload, inflammatory signalling, and tissue‐specific metabolic defects across multiple tissues.

In this context, targeting mitochondrial function is considered a promising strategy for improving insulin sensitivity [6, 8]. Current interventions focus mainly on exercise training and selected pharmacological or nutritional agents that modulate mitochondrial biogenesis and oxidative metabolism. However, interventions confined to single pathways or local tissues often fail to achieve coordinated and sustained metabolic improvement. In addition, some pharmacological strategies remain limited by long‐term safety concerns and translational challenges [28]. These observations further suggest that systemic recovery of insulin sensitivity depends on inter‐organ signalling mechanisms that coordinate mitochondrial adaptation across multiple metabolic tissues.

## 3 Extracellular Vesicles as Systemic Signalling Mediators in Exercise

### 3.1 Structural Features of Extracellular Vesicles

EVs are a heterogeneous population of membrane‐bound vesicles actively released by cells and widely present in body fluids, including blood, urine, and saliva. According to their biogenetic pathways and size, EVs are broadly classified into exosomes, microvesicles, and apoptotic bodies [29]. Although these subtypes differ in their biogenesis, they share a common structural feature. All EVs are enclosed by a phospholipid bilayer membrane that enables the stable encapsulation and delivery of diverse bioactive cargoes, including proteins, nucleic acids, lipids, and metabolism‐related molecules [30]. This vesicular architecture confers unique advantages for intercellular communication. Compared with freely soluble factors, EVs protect their internal cargo from enzymatic degradation in the circulation. They can also be efficiently taken up by recipient cells through membrane fusion, receptor‐mediated uptake, or endocytosis. These properties enable targeted delivery and functional regulation of signaling molecules. In addition, the cargo composition of EVs largely reflects the metabolic state and functional properties of their parent cells [31]. This feature allows EVs to transmit integrated biological information between different tissues. Based on these properties, EVs are now widely regarded as important mediators of intercellular and inter‐organ communication [32]. They are particularly well suited for coordinating energy metabolism and metabolic homeostasis, which require cooperation among multiple organs.

### 3.2 EV‐Mediated Crosstalk in Mitochondrial and Insulin‐Signalling Adaptation

A growing body of evidence suggests that EV‐mediated inter‐tissue communication plays an important role in diverse systemic physiological and pathological processes, including inflammation, immune regulation, tissue repair, and the maintenance of metabolic homeostasis [9]. In metabolic regulation, EVs can act in a paracrine or endocrine manner on neighbouring or distant tissues [33]. In this way, they integrate metabolic information and coordinate multi‐organ function at the whole‐body level.

Under insulin resistance–related conditions, the abundance, cellular origin, and cargo composition of circulating EVs are markedly altered. These changes suggest that EVs actively participate in the initiation and progression of metabolic disorders [34]. Under physiological or adaptive conditions, EVs derived from skeletal muscle, liver, or adipose tissue contribute to inter‐tissue metabolic coordination [34]. In contrast, under obesity or chronic metabolic dysfunction, aberrant EV signalling may amplify metabolic imbalance [35]. For example, skeletal muscle–derived EVs released during exercise or metabolic stress can be taken up by the liver and adipose tissue [36]. In recipient tissues, these EVs modulate energy metabolism and insulin sensitivity [37]. Conversely, EVs released from adipose tissue in obesity may be enriched in deleterious microRNAs or protein signals [37]. These cargos disrupt insulin signalling and exacerbate insulin resistance.

Thus, EV‐mediated inter‐tissue communication constitutes an important information network for regulating systemic metabolic balance [38]. This network shows strong context dependence under different physiological and pathological conditions. These properties make EVs a valuable entry point for understanding exercise‐induced systemic metabolic adaptation and the inter‐organ regulation of insulin resistance [39]. In the context of this review, the key issue is not simply whether EVs mediate inter‐organ communication, but how such communication converges on mitochondrial and insulin‐signalling endpoints [33]. Exercise‐induced EVs may link contracting skeletal muscle, liver, adipose tissue, vascular endothelium, and immune cells into a coordinated metabolic network. Within this network, skeletal muscle‐derived EVs may deliver mitochondrial regulatory proteins, metabolic enzymes, or miRNAs to local or distant metabolic tissues; adipose‐derived EVs may influence inflammatory tone, adipocyte insulin responsiveness, and lipid handling; liver‐derived EVs may participate in systemic substrate redistribution; and endothelial or blood cell‐derived EVs may regulate vascular function, immune activation, and tissue perfusion [37, 40, 41]. These coordinated effects may support mitochondrial remodelling, reduce metabolic stress, and create a systemic environment that favours insulin‐sensitive substrate utilization. Therefore, EV‐mediated crosstalk should be interpreted as a multi‐organ regulatory process that links exercise stimuli to mitochondrial adaptation and insulin signalling, rather than as a nonspecific form of intercellular communication.

### 3.3 Exercise‐Induced EVs as Metabolic Exerkine Carriers

The term “exerkines” refers to humoral signalling molecules released by multiple tissues in response to exercise. These molecules mediate the systemic effects of physical activity through autocrine, paracrine, or endocrine mechanisms [42]. Classical exerkines mainly include soluble factors and metabolites, such as interleukin‐6 (IL‐6), brain‐derived neurotrophic factor (BDNF), fibroblast growth factor 21 (FGF21), and lactate [43]. These factors play important roles in regulating inflammation and energy metabolism. However, they remain limited in explaining the complex and integrated systemic adaptations induced by exercise. This limitation reflects their short half‐lives, limited duration of action, and the inability of single factors to trigger coordinated metabolic remodelling across multiple organs.

Against this background, exercise‐induced EVs can be viewed as multicomponent metabolic exerkine carriers [43]. In contrast to classical soluble factors, EVs are physically structured signalling carriers. They are capable of packaging and delivering multiple classes of bioactive cargo simultaneously. Accumulating evidence indicates that both acute and chronic exercise increase the release of circulating EVs and reshape their microRNA, protein, and metabolism‐related cargo profiles [44, 45]. Owing to their lipid bilayer membrane, EVs show high stability in body fluids [46, 47]. They can also acquire tissue tropism through surface molecules. These properties enable multi‐layered metabolic regulation in a combinatorial manner.

Overall, despite heterogeneity in cellular origin and molecular composition, exercise‐induced EVs possess unique structural features and integrative signalling capacity. They should not be viewed merely as circulating biomarkers accompanying exercise. Instead, they function as important signalling mediators linking exercise stimuli to systemic metabolic adaptation. For the purposes of the present review, the importance of exercise‐induced EVs as metabolic exerkine carriers lies in their ability to package cargoes that are directly relevant to mitochondrial adaptation and insulin sensitivity [43]. These cargoes may include miRNAs that regulate PGC‐1α‐related mitochondrial programs, proteins associated with oxidative phosphorylation or substrate metabolism, and enzymes such as eNAMPT that may influence NAD^+^‐dependent metabolic signalling [48, 49]. In this sense, EVs differ from classical soluble exerkines not only because they are membrane‐enclosed carriers, but also because they can deliver coordinated cargo combinations capable of affecting mitochondrial biogenesis, oxidative metabolism, inflammatory tone, and insulin‐signalling pathways in target tissues [50]. This cargo‐based view provides a mechanistic basis for the subsequent discussion of how exercise‐induced EVs may regulate mitochondrial biogenesis and insulin sensitivity.

## 4 Exercise‐Induced Release of Extracellular Vesicles

### 4.1 Effects of Exercise Modalities on EV Release and Properties

A large body of evidence suggests that acute exercise can rapidly alter circulating EV abundance and phenotype, but the direction and magnitude of these changes depend strongly on exercise modality, EV subtype, sampling time, and metabolic state [51, 52]. During or immediately after aerobic endurance exercise, a single bout of moderate‐to‐high intensity activity, such as running or cycling, is often associated with a transient increase in circulating small EVs, particularly vesicles with diameters below 200 nm [50, 53]. These changes are accompanied by dynamic alterations in surface markers and internal cargo, indicating rapid activation of EV‐mediated systemic signalling. However, EV responses are not uniformly increased across all conditions. When measurements are performed several hours after exercise or on the following day, particularly under fasting or insulin‐stimulated conditions, circulating medium‐ to large‐size EVs may remain unchanged or decrease. For example, acute aerobic exercise has been reported to reduce fasting and insulin‐stimulated medium‐ to large‐size EVs in adults with obesity, including endothelial‐, leukocyte‐, platelet‐, and tetraspanin‐positive EV populations [54]. These findings indicate that acute exercise‐induced EV responses should be interpreted within a defined temporal and metabolic context.

Resistance exercise also induces EV remodelling, but its effects on EV abundance, size distribution, and surface phenotype appear more variable [55]. Some studies report changes in EV subpopulation composition and surface protein profiles [56], whereas others report no significant change or even a slight decrease in average EV size after resistance exercise [57]. These discrepancies likely reflect differences in exercise intensity, duration, training status, fasting or fed state, insulin‐stimulated conditions, and EV detection platforms. Therefore, rather than indicating inconsistency alone, the heterogeneous findings suggest that exercise‐induced EV release is dynamically regulated by both mechanical and metabolic cues.

The kinetics of EV release also differ between acute exercise and chronic training. Acute single‐bout exercise typically induces a transient increase in circulating EVs [58]. EV levels usually return to baseline within several hours to 1 day. In contrast, whether long‐term training leads to sustained changes in resting EV abundance or cargo composition remains controversial. Some studies suggest that repeated training reshapes basal EV cargo profiles [59]. Others report no significant change in baseline EV concentrations after weeks of training [60]. These discrepancies are likely related to differences in training modality, exercise intensity, duration, and individual metabolic status.

Overall, although different exercise modalities similarly increase EV release, they exert distinct effects on EV properties and cargo composition. EVs induced by aerobic endurance exercise tend to be enriched in molecules related to energy metabolism and mitochondrial adaptation. In contrast, resistance exercise–derived EVs may preferentially carry factors associated with muscle remodelling and tissue repair. These modality‐dependent differences provide important clues for understanding how exercise transmits specific metabolic signals through EVs.

### 4.2 Tissue Origins of Exercise‐Induced EVs

Skeletal muscle has long been considered a major source of exercise‐induced EVs [61]. This view is based on the large mass of skeletal muscle, its primary mechanical role during exercise, and its prominent endocrine functions. Previous studies have shown that exercise stimulates skeletal muscle to release multiple signalling molecules through vesicular pathways [42]. Some of these molecules enter the circulation in the form of EVs. Animal experiments and transgenic tracing studies provide direct evidence for skeletal muscle–derived EVs [40]. When circulating EVs are tracked using skeletal muscle–specific markers, such as α‐sarcoglycan, only approximately 1%–5% of plasma EVs display skeletal muscle signatures [61, 62]. This finding indicates that although skeletal muscle actively secretes EVs during exercise, only a small fraction enters the peripheral circulation [36]. A substantial proportion of muscle‐derived EVs may therefore act locally in a paracrine manner. Importantly, exercise‐induced circulating EVs do not originate from a single tissue. Instead, they reflect the coordinated response of multiple organs to exercise stimuli [63]. Surface marker profiling and flow cytometric analyses show that after strenuous exercise, circulating EVs carry diverse cellular signatures. These signatures include platelets, endothelial cells, lymphocytes, monocytes–macrophages, and antigen‐presenting cells. These observations indicate that blood cells and the vascular system are major contributors to circulating EVs during exercise. In particular, platelets and endothelial cells can rapidly release EVs in response to mechanical stress, shear forces, and neurohumoral signals [64]. Through this process, they participate in systemic signal transmission.

In addition to skeletal muscle and blood cells, the liver and adipose tissue also contribute to the exercise‐induced EV network [55]. Adipose tissue is now recognized as an active endocrine organ [65]. In obesity, adipose‐derived EVs are often enriched in pro‐inflammatory factors that promote insulin resistance [65]. Accumulating evidence suggests that regular exercise reshapes the cargo of adipose‐derived EVs [66]. This remodelling reduces pro‐inflammatory signals and enriches factors involved in fatty acid mobilization and anti‐inflammatory regulation. Similarly, during exercise, the liver maintains glucose homeostasis through glycogenolysis and gluconeogenesis [67]. These metabolic fluxes may be accompanied by the release of hepatokine‐rich EVs that participate in systemic energy redistribution. Together, these findings suggest that exercise‐induced circulating EVs arise from the coordinated secretion of multiple organs, including skeletal muscle, adipose tissue, and liver.

Despite this heterogeneity, not all EV sources contribute equally to metabolic regulation. EVs derived from metabolically active tissues, such as skeletal muscle, endothelium, and adipose tissue, are more likely to carry molecules related to energy metabolism, mitochondrial function, and insulin signaling. Although these EVs represent only a small fraction of total circulating EVs, their cargo composition and tissue targeting may confer biological effects that exceed their numerical abundance.

### 4.3 Source Heterogeneity and Signal Dominance

The heterogeneous origin of exercise‐induced EVs raises an important mechanistic question: whether mitochondrial biogenesis in metabolic tissues is driven mainly by a small but potent tissue‐specific EV subpopulation or by the net cargo profile of the heterogeneous circulating EV pool [61]. Current evidence does not support a simple single‐source model. Although skeletal muscle‐derived EVs represent only a small fraction of circulating EVs, their cargo composition, surface markers, and tissue‐targeting properties may allow them to exert biological effects disproportionate to their numerical abundance [68]. Such EV subpopulations may deliver muscle‐enriched miRNAs, metabolic enzymes, or mitochondrial regulatory proteins to recipient tissues and thereby directly influence mitochondrial adaptation [69, 70].

At the same time, the bulk circulating EV pool should not be regarded as functionally irrelevant [71]. Blood cell‐ and endothelial cell‐derived EVs, which constitute a large proportion of exercise‐induced circulating EVs, may shape the systemic metabolic environment by regulating vascular function, inflammation, immune activity, and tissue perfusion [71, 72]. These effects may indirectly facilitate mitochondrial biogenesis and insulin signaling in metabolic tissues. Therefore, the dominant EV‐mediated signal is likely not determined solely by numerical abundance, but by the combination of EV source, cargo potency, tissue tropism, uptake efficiency, and the coordinated change in the overall circulating EV profile.

Thus, we propose a dual‐level model. A small population of metabolically active, tissue‐derived EVs may provide cargo‐specific signals that directly regulate mitochondrial biogenesis, whereas the broader heterogeneous EV pool may create a permissive systemic environment for metabolic adaptation. Future studies using tissue‐specific EV tracing, single‐vesicle profiling, cargo‐specific manipulation, and cell‐type‐resolved EV isolation will be required to determine which EV populations are dominant drivers of mitochondrial biogenesis and insulin sensitivity in vivo [56, 73].

### 4.4 Changes in the Cargo Composition of Exercise‐Induced EVs

Exercise induces pronounced remodelling of EV cargo composition, with preferential enrichment of molecules related to energy metabolism and mitochondrial function [74]. Proteomic and nucleic acid–based analyses consistently show that, compared with the resting state, the protein, microRNA (miRNA), and metabolic enzyme profiles of circulating EVs are markedly altered after exercise [75]. These findings indicate that exercise not only modulates EV abundance and origin but also reshapes their molecular cargo.

At the protein level, multiple studies report enrichment of mitochondrial‐related proteins in EVs isolated after endurance exercise. These proteins include subunits of oxidative phosphorylation complexes and enzymes involved in substrate oxidation and energy conversion. Quantitative proteomic analyses further show that acute aerobic exercise promotes the release of many metabolism‐related proteins into the circulation via EVs [50, 76]. A substantial proportion of these proteins are closely associated with mitochondrial structure and function. In addition to proteins, exercise markedly influences the miRNA cargo of EVs. Both acute and chronic exercise reshape the expression profiles of multiple metabolism‐related miRNAs in circulating EVs [77]. For example, skeletal muscle–enriched miRNAs, such as miR‐133a/b, members of the miR‐23 family, and miR‐696, show altered levels after exercise [78]. These miRNAs have been implicated in the regulation of energy metabolism and mitochondrial gene expression. When delivered to metabolically active target tissues, such as skeletal muscle, liver, adipose tissue, and vascular endothelium, these miRNAs may contribute to systemic metabolic adaptation [33]. Exercise‐induced EVs also carry enzymes that directly regulate cellular energy metabolism [79, 80]. Notably, exercise increases the abundance of extracellular nicotinamide phosphoribosyltransferase (eNAMPT) in circulating EVs [81]. As a rate‐limiting enzyme in the NAD^+^ salvage pathway, eNAMPT delivered via EVs may play an important role in coordinating metabolic regulation across multiple tissues [81, 82].

Overall, by reshaping the protein, miRNA, and metabolic enzyme cargo of EVs, exercise confers a distinct metabolic adaptive signature on circulating EVs. These cargo molecules are closely linked to mitochondrial function and energy metabolism. They provide a potential molecular basis for EV‐mediated inter‐organ metabolic signalling. How these EV cargoes regulate mitochondrial biogenesis and metabolic adaptation in specific metabolic target tissues and cells will be discussed in the following section.

## 5 Molecular Mechanisms Linking Exercise‐Induced EVs, Mitochondrial Adaptation, and Insulin Sensitivity

**FIGURE 1:**
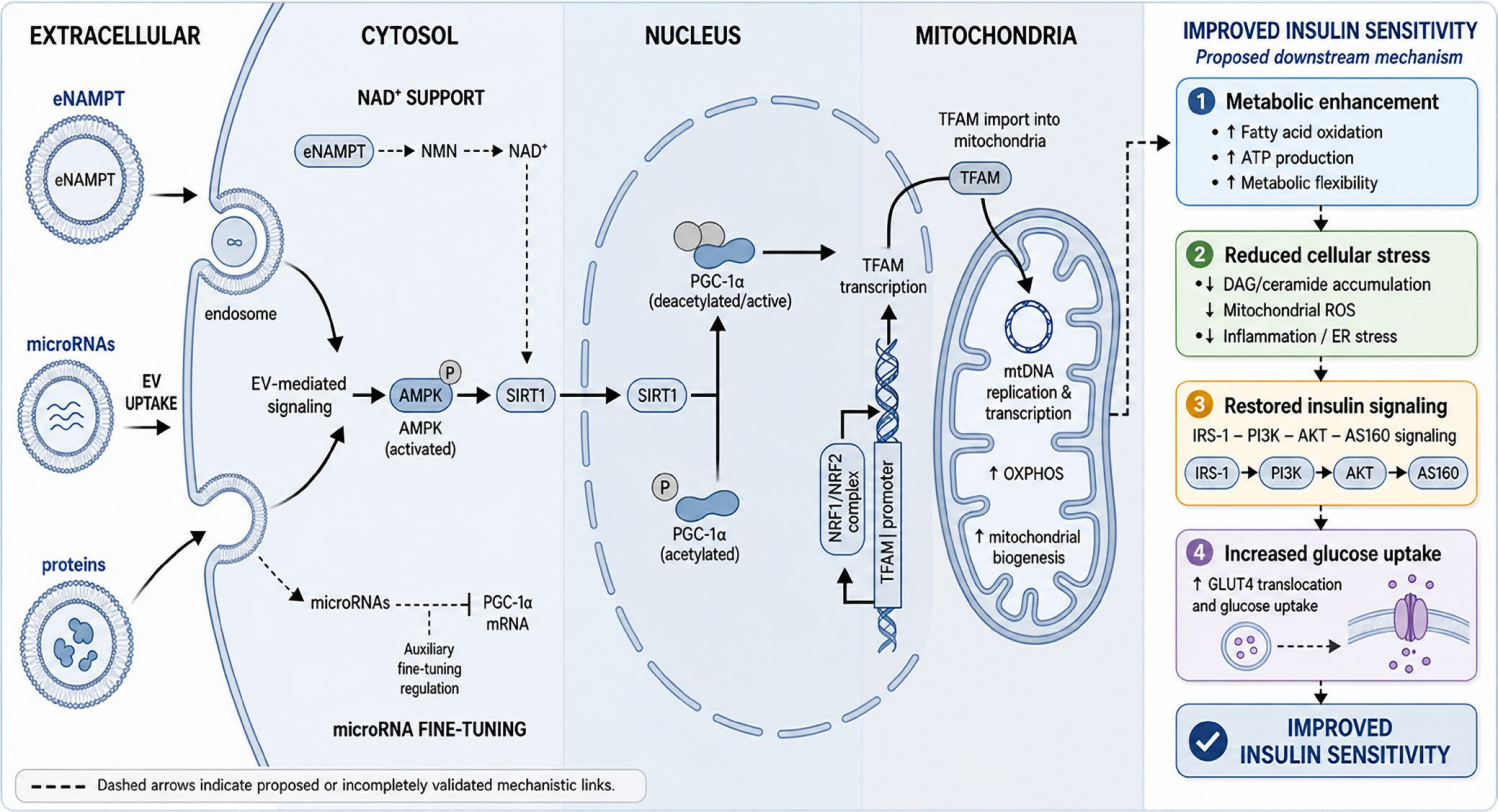
Proposed mechanisms linking exercise‐induced extracellular vesicles to mitochondrial adaptation and improved insulin sensitivity. Exercise‐induced extracellular vesicles (EVs) may deliver multiple classes of bioactive cargoes, including extracellular nicotinamide phosphoribosyltransferase (eNAMPT), microRNAs, and proteins, to metabolically active recipient cells. After EV uptake, these cargoes may influence cytosolic energy‐sensing and metabolic pathways, including AMP‐activated protein kinase (AMPK), nicotinamide adenine dinucleotide (NAD^+^) metabolism, and sirtuin 1 (SIRT1)‐related signalling. Activation of the AMPK–SIRT1–peroxisome proliferator‐activated receptor‐γ coactivator 1α (PGC‐1α) axis may promote mitochondrial biogenesis through nuclear respiratory factor 1/2 (NRF1/NRF2) and mitochondrial transcription factor A (TFAM), thereby supporting mitochondrial DNA replication, oxidative phosphorylation (OXPHOS), ATP production, and metabolic flexibility. In parallel, EV‐associated microRNAs may fine‐tune mitochondrial regulatory programs in a cargo‐specific and context‐dependent manner. These mitochondrial adaptations may improve insulin sensitivity by enhancing fatty acid oxidation, reducing diacylglycerol/ceramide accumulation, mitochondrial reactive oxygen species (ROS), inflammation, and endoplasmic reticulum (ER) stress, and restoring insulin receptor substrate 1 (IRS‐1)–phosphoinositide 3‐kinase (PI3K)–AKT–AS160 signalling and glucose transporter 4 (GLUT4)‐mediated glucose uptake. Dashed arrows indicate proposed or incompletely validated mechanistic links.

Accumulating evidence suggests that exercise‐induced EVs do not exert their metabolic effects through a single signalling molecule. Instead, they may modulate mitochondrial biogenesis–related programs in metabolically active recipient tissues through a multi‐layered and integrated signalling network [80]. Based on current exercise‐related and metabolic EV studies, relevant target cells may include skeletal muscle cells/myoblasts or myotubes, hepatocytes, adipocytes, endothelial cells, and, in cardiovascular disease‐related contexts, cardiomyocytes [41, 70]. Based on available evidence, exercise‐induced or contraction‐related EVs may influence mitochondrial adaptation in recipient cells through several partially characterized mechanisms, including EV‐associated modulation of energy/NAD^+^‐related signalling, delivery of metabolic enzymes such as NAMPT, and miRNA‐mediated regulation of mitochondrial and metabolic programs [48, 83]. In parallel, metabolic support pathways and post‐transcriptional regulatory mechanisms likely act as auxiliary modules that amplify and stabilize exercise‐induced mitochondrial remodelling (Figure 1).

### 5.1 The AMPK–SIRT1–PGC‐1α Signalling Axis

The AMPK–SIRT1–PGC‐1α signalling axis is a well‐established energy‐sensing network that links cellular metabolic stress to mitochondrial biogenesis [7]. AMPK functions as a cellular energy sensor and is activated under conditions of increased AMP/ATP or ADP/ATP ratios. Activated AMPK can promote mitochondrial adaptation by enhancing fatty acid oxidation, inhibiting anabolic lipid synthesis, and regulating PGC‐1α activity. SIRT1, an NAD^+^‐dependent deacetylase, can further modulate this pathway by deacetylating PGC‐1α and enhancing its transcriptional coactivator activity. Through cooperation with NRF1, NRF2, and TFAM, activated PGC‐1α drives the expression of nuclear‐encoded mitochondrial proteins and supports mitochondrial DNA replication and respiratory chain assembly [18, 84].

Experimental studies suggest that exposure of defined metabolic target cells, such as skeletal muscle cells/myotubes, hepatocytes, adipocytes, endothelial cells, or cardiomyocytes, to exercise‐derived or exercise‐related EVs may be associated with increased AMPK phosphorylation and downstream mitochondrial adaptive responses [48, 70]. However, the precise mechanisms by which EVs activate AMPK, and whether they directly regulate SIRT1 in specific target cell types, remain incompletely defined. Current evidence suggests that EVs may influence this signalling network indirectly by delivering proteins, enzymes, metabolites, or regulatory RNAs that alter cellular energy balance, NAD^+^ metabolism, oxidative stress, calcium‐dependent signalling, or upstream kinase activity [48, 85]. For example, exercise‐induced EVs can carry NAMPT, which may modulate NAD^+^ availability in target cells and thereby create a metabolic context permissive for NAD^+^‐dependent SIRT1 activity and PGC‐1α activation. However, this should not be interpreted as definitive evidence that EVs directly activate SIRT1 as a primary target. Other EV cargoes may affect mitochondrial function or substrate metabolism, creating energetic stress signals that converge on AMPK phosphorylation. Thus, EV‐associated modulation of the AMPK–SIRT1–PGC‐1α pathway should be interpreted as a multi‐step and context‐dependent process rather than a direct single‐cargo effect. Further studies are needed to identify the specific EV cargoes, upstream kinases, recipient cell types, and causal signalling events responsible for AMPK and SIRT1‐related regulation.

### 5.2 The NRF1/NRF2–TFAM Execution Module

Following activation of PGC‐1α, the execution phase of mitochondrial biogenesis is mediated by NRF1, NRF2, and TFAM [86]. PGC‐1α enhances the transcriptional activity of NRF1 and NRF2. In this way, it promotes the expression of nuclear‐encoded mitochondrial proteins and upregulates TFAM. After translocation into mitochondria, TFAM participates directly in mitochondrial DNA replication, transcription, and structural maintenance. Through these processes, TFAM ensures coordinated expression of nuclear and mitochondrial genomes during respiratory chain protein synthesis. For example, EVs released from skeletal muscle after chronic contractile activity have been shown to increase TFAM expression, mitochondrial respiratory chain protein abundance, oxygen consumption, and ATP production in recipient myoblasts [70]. These findings suggest that contraction‐related skeletal muscle‐derived EVs may support mitochondrial biogenesis and respiratory function in defined muscle‐lineage target cells, thereby contributing to mitochondrial adaptation and metabolic remodelling.

### 5.3 The eNAMPT–NAD ^+^ Metabolic Support Pathway

In addition to signalling proteins, metabolic enzymes carried by exercise‐induced EVs may provide metabolic support for mitochondrial adaptation [48, 50]. Among these enzymes, extracellular nicotinamide phosphoribosyltransferase (eNAMPT) has emerged as a potential link between EV signalling and NAD^+^ metabolism [87]. Evidence indicates that exercise can increase the release of NAMPT in circulating EVs and alter NAD^+^‐related metabolic activity in recipient cells. These findings support the possibility that EV‐associated NAMPT may influence cellular NAD^+^ availability.

Mechanistically, NAMPT is the rate‐limiting enzyme in the NAD^+^ salvage pathway, catalysing the conversion of nicotinamide to nicotinamide mononucleotide [87]. Increased NAD^+^ availability could, in principle, support NAD^+^‐dependent enzymes such as SIRT1 and SIRT3 [84, 88]. SIRT1 may promote PGC‐1α deacetylation and mitochondrial gene regulation, whereas SIRT3 regulates the acetylation status and activity of mitochondrial proteins [18, 89]. However, in the context of exercise‐induced EVs, the complete causal sequence linking EV‐associated NAMPT to NAD^+^ elevation, SIRT activation, PGC‐1α regulation, and mitochondrial biogenesis has not been fully established. Therefore, this pathway should be interpreted as a plausible metabolic support mechanism rather than a definitively proven EV‐driven mitochondrial biogenesis cascade.

Future studies are needed to determine whether EV‐delivered NAMPT is sufficient and necessary to increase intracellular NAD^+^ levels in specific target cells, such as skeletal muscle cells/myotubes, hepatocytes, adipocytes, endothelial cells, or cardiomyocytes. It will also be important to define whether SIRT1/SIRT3 activation and PGC‐1α‐dependent mitochondrial remodelling occur downstream of EV‐associated NAMPT under different exercise modalities, donor metabolic states, and recipient cell contexts.

### 5.4 miRNA‐Mediated Post‐Transcriptional Regulation

Beyond core signalling and metabolic support pathways, microRNAs carried by exercise‐induced EVs may provide post‐transcriptional regulation of mitochondrial adaptation [83]. miRNAs, including miR‐23 family members and miR‐696, have been reported to suppress PGC‐1α expression or activity under resting or metabolic stress conditions, thereby limiting mitochondrial biogenesis [49, 90]. However, exercise‐induced changes in EV‐associated miRNAs are miRNA‐specific and context‐dependent, and their direction of change should be clearly considered [91]. For inhibitory miRNAs that target PGC‐1α‐related mitochondrial programs, a reduction in their EV‐associated abundance could theoretically relieve suppression of mitochondrial biogenesis, whereas an increase may further attenuate mitochondrial adaptation [49]. Therefore, EV‐derived miRNAs should not be interpreted as uniformly pro‐mitochondrial signals. Rather, they may either support or restrain mitochondrial remodelling depending on the specific miRNA cargo, exercise modality, donor metabolic status, and target cell type [83].

Compared with the core mitochondrial regulatory pathways discussed above, EV‐associated miRNAs likely act as modulators of the amplitude and precision of mitochondrial adaptation rather than as independent drivers [92]. Future studies should define the direction of exercise‐induced changes in individual EV miRNAs and determine their causal effects on mitochondrial biogenesis in defined target cells, such as skeletal muscle cells/myotubes, hepatocytes, adipocytes, endothelial cells, or cardiomyocytes.

### 5.5 Metabolic and Signalling Links Between EV‐Induced Mitochondrial Adaptation and Insulin Sensitivity

Although exercise‐induced EVs are increasingly linked to mitochondrial biogenesis, the mechanistic connection between EV‐induced mitochondrial adaptation and improved insulin sensitivity should not be interpreted simply as “better mitochondria lead to better metabolism” [6, 11]. Instead, EV‐mediated mitochondrial remodelling may improve insulin sensitivity through both metabolic and signalling‐based mechanisms.

From a metabolic perspective, enhanced mitochondrial biogenesis and oxidative phosphorylation increase the oxidative capacity of recipient tissues [6]. In skeletal muscle, improved mitochondrial function may promote fatty acid oxidation and metabolic flexibility, thereby reducing the accumulation of lipid intermediates that interfere with insulin‐stimulated glucose uptake [24]. In hepatocytes and adipocytes, enhanced mitochondrial oxidative metabolism may similarly limit lipid overload and reduce the accumulation of diacylglycerols and ceramides [2]. These lipid species can activate stress‐sensitive kinases and impair insulin receptor substrate signaling [11]. Therefore, by reducing lipotoxic stress, EV‐induced mitochondrial adaptation may help preserve IRS‐1/IRS‐2–PI3K–AKT signaling and improve tissue insulin responsiveness [93].

Mitochondrial adaptation may also improve insulin sensitivity by attenuating oxidative and inflammatory stress [21]. Mitochondrial dysfunction is frequently associated with excessive mitochondrial ROS production, ER stress, and activation of inflammatory pathways, all of which can impair insulin signaling in metabolic tissues. By improving mitochondrial quality, respiratory efficiency, and substrate oxidation, exercise‐induced EVs may reduce mitochondrial ROS generation and inflammatory signaling [6, 21]. This effect may be particularly relevant in liver and adipose tissue, where chronic lipid accumulation and low‐grade inflammation contribute to systemic insulin resistance.

In addition to these metabolic effects, EV cargoes may directly regulate insulin‐related signalling networks [94]. Exercise‐induced EVs carry proteins, miRNAs, and metabolic enzymes that can influence AMPK activity, NAD + ‐dependent sirtuin signalling, and nutrient‐sensitive pathways such as mTOR [95]. These pathways interact with insulin signalling at multiple levels. For example, AMPK activation may enhance glucose uptake and fatty acid oxidation, while altered mTOR signalling can influence insulin receptor substrate function and downstream AKT activity [96]. EV‐associated miRNAs may also modulate the expression of genes involved in mitochondrial function, lipid metabolism, inflammation, and insulin signal transduction [97]. Thus, EV‐mediated improvements in insulin sensitivity may arise not only from enhanced mitochondrial substrate oxidation, but also from cargo‐dependent modulation of insulin signalling components, including IRS proteins, AKT, AS160, and GLUT4 trafficking.

Taken together, EV‐induced mitochondrial adaptation may act as a mechanistic bridge between exercise‐induced inter‐organ communication and improved insulin sensitivity [98]. In this model, exercise‐induced EVs promote mitochondrial biogenesis and oxidative metabolism, which reduces lipotoxicity, mitochondrial ROS, ER stress, and inflammatory signalling [99]. At the same time, EV cargoes may directly or indirectly modulate AMPK–mTOR crosstalk and insulin signalling pathways [95, 100]. Therefore, the improvement in insulin sensitivity is likely driven by the combined effects of metabolic remodelling and signalling regulation rather than by mitochondrial biogenesis alone. Nevertheless, direct evidence linking specific EV cargoes, mitochondrial changes, and downstream insulin signalling remains limited. Future studies should combine EV transfer or inhibition approaches with tissue‐specific measurements of mitochondrial flux, DAG and ceramide levels, ROS production, IRS–AKT–AS160 signalling, GLUT4 translocation, and glucose uptake.

## 6 Functional Evidence for Exercise‐Induced Extracellular Vesicles in the Regulation of Insulin Sensitivity

The mechanisms described above suggest that EV‐induced mitochondrial adaptation may improve insulin sensitivity by enhancing oxidative metabolism and reducing metabolic stress. Functional studies provide support for this model by showing that exercise‐induced EVs or EV‐associated cargoes are linked to improvements in glucose tolerance, lipid handling, and insulin‐responsive tissue function [40, 91].

In recent years, a series of animal experiments show that when EV biogenesis or release induced by exercise is pharmacologically or genetically inhibited, the beneficial effects of exercise on insulin resistance and metabolic homeostasis are partially attenuated [94, 101]. These findings suggest that EVs represent an important functional component of exercise‐induced metabolic adaptation.

In high‐fat diet–induced obese and insulin‐resistant mouse models, chronic exercise training has been reported to improve selected metabolic outcomes, including glucose tolerance, serum lipid profiles, and hepatic lipid accumulation [102]. Separately, EV inhibition studies suggest that blocking EV release, for example through pharmacological inhibition of neutral sphingomyelinase activity, may attenuate selected exercise‐associated metabolic benefits in specific experimental settings [40]. However, this should not be interpreted as evidence that EV inhibition blunts all cardiometabolic adaptations induced by exercise. Rather, the affected outcomes appear to depend on the disease model, exercise protocol, EV inhibition strategy, and metabolic endpoints assessed. These results indicate that EVs are not merely passive byproducts accompanying exercise, but actively participate in supporting the protective effects of exercise on metabolic homeostasis. Similar functional evidence has been obtained in other metabolic disease models. Aerobic training reshapes the microRNA cargo profiles of circulating EVs in obese mice, with reductions in miR‐122, miR‐192, and miR‐22, microRNAs previously implicated in impaired insulin sensitivity and dysregulated lipid metabolism in the liver and adipose tissue [103]. In parallel with these EV cargo changes, trained mice exhibit reduced adipocyte size, increased expression of mitochondrial function–related genes, and decreased hepatic steatosis and inflammation. In type 2 diabetic (db/db) mouse models, regular low‐intensity exercise increases the abundance of circulating EVs enriched in miR‐445 [104]. This microRNA suppresses myocardial fibrosis by downregulating MMP9, indicating that exercise‐induced EVs may contribute not only to improvements in insulin resistance, but also to the modulation of metabolic complications.

Notably, several recent human studies provide indirect support for the metabolic regulatory roles of exercise‐induced EVs under physiological conditions [105]. In overweight individuals, Doncheva and colleagues reported that acute exercise and longer‐term exercise training induced miRNA‐specific changes in plasma EV cargo, with some EV‐associated miRNAs increasing and others decreasing rather than showing a uniform directional response [91]. These miRNA changes were associated with metabolic parameters, including insulin sensitivity and adipose tissue inflammatory markers, although the study was observational and does not establish a causal role for EV miRNAs in mediating exercise‐induced metabolic improvements. Although these observational studies cannot establish causality, they partially bridge the gap between animal models and clinical reality and provide preliminary human evidence supporting the translational relevance of exercise‐induced EVs in metabolic health.

EV transfer experiments further strengthen the functional relevance of exercise‐induced EVs in the regulation of insulin sensitivity [98]. In several animal studies, EVs isolated from exercised mice, particularly skeletal muscle‐derived exosome‐like EVs generated under exercise or contraction‐related conditions, were administered to sedentary or metabolically impaired recipient mice. These experiments were designed to test whether EVs could reproduce selected metabolic benefits of exercise in the absence of physical activity. Administration of exercise‐derived EVs has been reported to improve glucose tolerance and reduce the area under the glucose tolerance curve in high‐fat diet‐induced obese mice [106, 107]. These effects are accompanied by decreases in serum triglyceride and total cholesterol levels, suggesting improved systemic metabolic regulation. Similar metabolic benefits have also been observed in ApoE^−^/^−^ atherosclerosis models. Tissue distribution analyses indicate that exercise‐induced or muscle‐derived EVs can be taken up by metabolic organs, including the liver and spleen [40, 66]. In relevant metabolic disease models, exercise‐related or muscle‐derived EV administration has also been associated with reduced hepatic lipid accumulation and improved systemic metabolic parameters. However, whether these hepatic effects are directly mediated by EV uptake in the liver remains to be further established.

These observations indicate that exercise‐induced EVs possess metabolic regulatory capacity, but they do not exclude the contribution of other exercise‐activated systemic factors. Importantly, the metabolic improvements observed in EV transfer experiments usually represent a partial recapitulation of exercise effects [52]. This finding suggests that exercise‐induced EVs are not the sole mediators of exercise‐induced improvements in insulin resistance. Instead, they may function as an important signalling module acting in concert with classical exerkines and neuroendocrine regulatory mechanisms. Therefore, even in the absence of mechanical exercise stimuli, transferred EVs can reproduce selected aspects of exercise‐associated metabolic improvement, including enhanced glucose handling and mitochondrial‐related adaptation in specific experimental models [108, 109]. These results support a causal, but context‐dependent, contribution of exercise‐induced EVs to metabolic regulation.

Collectively, in vivo functional evidence derived from EV inhibition and transfer experiments indicates that exercise‐induced EVs amplify and integrate exercise‐induced metabolic adaptation. They deliver metabolic signals related to mitochondrial biogenesis and energy metabolism across multiple tissues. These findings provide functional support for the molecular mechanisms described above and reinforce the biological significance of the “exercise–EV–mitochondrial adaptation–insulin sensitivity” framework in metabolic diseases.

## 7 Critical Assessment of EV Causality and Translational Challenges

Although mechanistic and functional studies support the involvement of exercise‐induced EVs in mitochondrial adaptation and insulin sensitivity, the strength of causal evidence and the feasibility of translating these findings into EV‐based interventions remain important unresolved issues [98]. Therefore, this section critically discusses two major limitations: first, the difficulty of distinguishing correlation from causality in animal and human studies; and second, the translational gap between physiological EV flux during exercise and experimental or therapeutic EV administration.

### 7.1 Correlation, Causality, and Challenges in Isolating EV Effects In Vivo

Human studies provide valuable translational clues but remain largely observational. Exercise‐induced changes in circulating EV abundance or cargo composition have been associated with improved insulin sensitivity, inflammatory markers, or metabolic profiles [91]. However, these associations do not establish causality because exercise simultaneously changes soluble exerkines, metabolites, neuroendocrine signals, immune activity, blood flow, and tissue metabolic fluxes [9].

EV inhibition studies provide evidence for a partially necessary role of EVs, because blocking EV biogenesis or release can attenuate exercise‐induced improvements in glucose tolerance, lipid metabolism, or tissue function [40]. However, these approaches are not completely EV‐specific. For example, pharmacological inhibitors such as GW4869 may also affect sphingolipid metabolism and other vesicle‐independent processes [110]. Therefore, inhibition studies support partial necessity, but they cannot prove that EVs are the sole required mediators.

EV transfer studies provide evidence for partial sufficiency, because EVs isolated from exercised animals can reproduce selected metabolic benefits in sedentary or metabolically impaired recipients [40]. Nevertheless, these effects usually represent only a partial recapitulation of exercise benefits [66]. EV administration cannot reproduce the full physiological context of exercise, including muscle contraction, substrate flux, hormonal responses, soluble exerkines, and neural regulation.

Taken together, current evidence supports exercise‐induced EVs as important contributors to metabolic adaptation, rather than as the sole or fully sufficient mediators. Their role is best understood as one signalling module within a broader exercise‐responsive network. Future studies should combine tissue‐specific EV tracing, cargo‐specific loss‐ and gain‐of‐function approaches, standardized EV isolation, and parallel assessment of soluble factors and metabolic fluxes.

### 7.2 Translational Challenges: Physiological EV Flux, Experimental Dosing, and Pharmacokinetics

A major translational challenge is the gap between physiological EV flux during exercise and the experimental or therapeutic administration of purified EVs [111]. During exercise, EV release, tissue uptake, and clearance occur dynamically and simultaneously. In contrast, most transfer studies administer purified EVs as bolus injections, which may create exposure patterns that differ substantially from physiological exercise‐induced EV signalling [111].

Reported EV doses vary widely across studies and are often expressed using different units, including particle number, protein amount, or injection volume [112]. For example, some studies used microgram‐based EV doses, whereas others reported particle numbers such as 10^8–10^10 particles per injection [13, 60]. These values are difficult to compare directly with endogenous EV release during exercise because circulating EV concentration reflects the balance between release, uptake, redistribution, and clearance rather than total EV production.

This distinction has important pharmacokinetic implications. Systemically administered EVs are rapidly distributed and cleared, particularly by the liver, spleen, and other components of the reticuloendothelial system [113]. Therefore, a bolus injection may not reproduce the gradual, repeated, and tissue‐specific EV exposure induced by exercise. Delivery efficiency to metabolic target organs may also be limited by EV heterogeneity, surface markers, tissue tropism, uptake efficiency, and clearance rate.

Thus, exercise‐induced EVs should not yet be viewed as a direct replacement for exercise. At present, they are better understood as mechanistic tools and potential templates for exercise‐mimetic strategies. Future translational studies should standardize EV dose reporting, compare administered EV doses with endogenous exercise‐induced EV kinetics, define dose–response relationships, and evaluate biodistribution, clearance, safety, tissue targeting, and manufacturing scalability.

## 8 Conclusion and Perspectives

Accumulating evidence over recent years suggests that exercise‐induced EVs represent an important signalling interface linking local exercise stimuli to systemic metabolic adaptation. Beyond the traditional view that emphasizes intrinsic metabolic remodelling within skeletal muscle, exercise‐released EVs can circulate stably in body fluids and be taken up by multiple metabolic organs. Through this process, they facilitate the coordination of mitochondrial biogenesis and functional remodelling at the whole‐body level. By delivering proteins, metabolic enzymes, and microRNAs associated with energy sensing and mitochondrial regulation, exercise‐induced EVs are linked to the activation of key signalling pathways, including the AMPK–SIRT1–PGC‐1α, NRF1/NRF2–TFAM, and eNAMPT–NAD^+^–Sirtuin axes. Through these pathways, EVs may contribute to mitochondrial DNA replication, respiratory chain protein synthesis, and oxidative metabolic capacity. Together, these coordinated actions provide a plausible cross‐organ molecular basis for exercise‐associated improvements in insulin sensitivity and the maintenance of metabolic homeostasis.

Based on current mechanistic studies, animal models, and emerging human observations, we propose an integrative “exercise–EV–mitochondrial adaptation–insulin sensitivity” framework. In this framework, exercise acts as an initiating stimulus that induces the release of EVs with specific cargo signatures from skeletal muscle and other tissues. These EVs mediate inter‐organ communication through the circulation and are functionally linked to the activation of mitochondrial biogenesis programs in target organs, including the liver, adipose tissue, and skeletal muscle. The resulting changes in mitochondrial content, function, and metabolic flexibility may enhance fatty acid oxidation and glucose utilization, alleviate lipotoxic and oxidative stress, and thereby contribute to improved insulin signaling in a context‐dependent manner. Through these effects, EV‐mediated signaling may contribute to the restoration of systemic metabolic homeostasis. This framework integrates molecular mechanisms, in vivo functional evidence, and human associative data and provides a unified conceptual basis for understanding the systemic metabolic benefits of exercise.

From a translational perspective, exercise‐induced EVs are theoretically attractive as potential “exercise‐mimetic” mediators, particularly for individuals who are unable to engage in sufficient physical activity. Compared with single‐target pharmacological agents, EVs carry multiple classes of bioactive molecules and therefore have the potential to coordinately regulate mitochondrial function, energy metabolism, and insulin signalling. Nevertheless, current research on exercise‐induced EVs remains largely at the stage of mechanistic exploration and animal experimentation, and their clinical application faces substantial challenges. EVs derived from different tissues, exercise modalities, and isolation protocols exhibit marked heterogeneity in size, membrane composition, and cargo profiles, which complicates standardization and cross‐study comparability. Moreover, systemically administered EVs are rapidly cleared by the reticuloendothelial system, particularly by the liver and spleen, which limits their delivery efficiency to metabolic target organs. In addition, non‐target cargos carried by EVs may elicit unpredictable biological effects, and their potential immunogenicity and long‐term safety remain insufficiently characterized. At present, there is still no consensus on standardized protocols for EV preparation, quality control, and dose evaluation, all of which represent major barriers to clinical translation.

Despite these limitations, current evidence delineates several clear directions for future research. Under rigorously controlled exercise prescriptions, it will be essential to define how different exercise modalities, exercise intensity and duration, and population characteristics shape EV abundance, tissue origin, and cargo composition. Future studies should explicitly consider population heterogeneity, including sex, age, developmental stage, hormonal status, and baseline metabolic health. In particular, potential differences between children, young adults, older adults, and frail populations remain poorly understood, and hormonal transitions such as menopause and andropause may influence EV release, cargo profiles, mitochondrial function, inflammatory tone, and insulin sensitivity. Diet should also be considered as an important contextual modifier, because dietary composition, caloric balance, fasting or postprandial state, and obesity‐related nutrient excess may alter both exercise‐induced EV responses and the metabolic responsiveness of recipient tissues. Therefore, future human and animal studies should clearly report and, where possible, control or stratify by sex, age, hormonal status, dietary background, feeding state, and baseline metabolic phenotype. It will also be important to identify the core EV effector molecules with causal relevance to mitochondrial biogenesis and insulin sensitivity. In parallel, EV inhibition and transfer experiments should be extended to additional models of metabolic disease to further establish necessity and sufficiency. Multi‐omics approaches should be employed to delineate the cross‐organ regulatory networks mediated by EVs. From a translational standpoint, the development of engineered EVs or optimized delivery strategies to enhance tissue targeting, reduce immunogenicity, and improve pharmacokinetic properties, together with standardized manufacturing pipelines, will be crucial steps toward clinical application.

Overall, research on exercise‐induced EVs provides a new systems‐level perspective on how exercise improves metabolic health and offers a conceptual foundation for the development of EV‐based metabolic interventions. As mechanistic insights deepen and technological platforms advance, elucidating EV‐mediated mitochondrial remodelling may ultimately clarify key molecular components of exercise benefits and open new avenues for precision interventions against insulin resistance and related metabolic diseases.

## Author Contributions

**Jian Wang:** conceptualization, writing – review and editing, writing – original draft, project administration. **Lei Wu:** conceptualization, writing – review and editing, writing – original draft, project administration, supervision.

## Funding

The authors have nothing to report.

## Ethics Statement

The authors have nothing to report.

## Consent

The authors have nothing to report.

## Conflicts of Interest

The authors declare no conflicts of interest.

## Data Availability

Data sharing not applicable to this article as no datasets were generated or analysed during the current study.
